# Subjective aggression during alcohol and cannabis intoxication before and after aggression exposure

**DOI:** 10.1007/s00213-016-4371-1

**Published:** 2016-07-15

**Authors:** E. B. De Sousa Fernandes Perna, E. L. Theunissen, K. P. C. Kuypers, S. W. Toennes, J. G. Ramaekers

**Affiliations:** 1 Department Neuropsychology and Psychopharmacology, Faculty of Psychology and Neuroscience, Maastricht University, P.O. Box 616, 6200 MD Maastricht, The Netherlands; 2 Department of Forensic Toxicology, Institute of Legal Medicine, Goethe University of Frankfurt, Frankfurt, Germany

**Keywords:** Intoxicated aggression, Alcohol, Cannabis, Cortisol, Testosterone

## Abstract

**Rationale:**

Alcohol and cannabis use have been implicated in aggression. Alcohol consumption is known to facilitate aggression, whereas a causal link between cannabis and aggression has not been clearly demonstrated.

**Objectives:**

This study investigated the acute effects of alcohol and cannabis on subjective aggression in alcohol and cannabis users, respectively, following aggression exposure. Drug-free controls served as a reference. It was hypothesized that aggression exposure would increase subjective aggression in alcohol users during alcohol intoxication, whereas it was expected to decrease subjective aggression in cannabis users during cannabis intoxication.

**Methods:**

Heavy alcohol (*n* = 20) and regular cannabis users (*n* = 21), and controls (*n* = 20) were included in a mixed factorial study. Alcohol and cannabis users received single doses of alcohol and placebo or cannabis and placebo, respectively. Subjective aggression was assessed before and after aggression exposure consisting of administrations of the point-subtraction aggression paradigm (PSAP) and the single category implicit association test (SC-IAT). Testosterone and cortisol levels in response to alcohol/cannabis treatment and aggression exposure were recorded as secondary outcome measures.

**Results:**

Subjective aggression significantly increased following aggression exposure in all groups while being sober. Alcohol intoxication increased subjective aggression whereas cannabis decreased the subjective aggression following aggression exposure. Aggressive responses during the PSAP increased following alcohol and decreased following cannabis relative to placebo. Changes in aggressive feeling or response were not correlated to the neuroendocrine response to treatments.

**Conclusions:**

It is concluded that alcohol facilitates feelings of aggression whereas cannabis diminishes aggressive feelings in heavy alcohol and regular cannabis users, respectively.

**Electronic supplementary material:**

The online version of this article (doi:10.1007/s00213-016-4371-1) contains supplementary material, which is available to authorized users.

## Introduction

Alcohol and cannabis are among the most frequently used drugs worldwide (EMCDDA 2012). The elicitation of aggressive behavior following alcohol consumption, also called “intoxicated aggression,” has been frequently reported on a global scale (Murdoch et al. 1990). Cannabis intoxication, however, does not typically lead to aggression in most individuals (Hoaken and Stewart 2003), but it might increase or facilitate aggression in certain subgroups (i.e., violent offenders, clinical population) (Cherek et al. 1993). However, not everybody who uses alcohol or cannabis engages in aggressive behaviors (Heinz et al. 2001; Kopak et al. 2014; Lammers et al. 2014). A clear relationship between alcohol, drugs, and intoxicated aggression is neither linear nor invariant. Some drugs can facilitate aggressive behavior through their direct pharmacological effects during intoxication, through neurotoxic effects caused by chronic drug use over time or through withdrawal effects during abstinence (Hoaken and Stewart 2003).

The relation between alcohol consumption and aggression has been well established. Experimental studies on aggression have demonstrated that acute doses of alcohol facilitate aggressive behavior in a dose-related manner as assessed by vocal recordings and questionnaires (Bushman and Cooper 1990; Ito et al. 1996). Studies using laboratory-based measures of aggression have generally found that aggression was higher in participants who were intoxicated compared to those who received no alcohol (for a review see Giancola and Chermack, 1998). Longitudinal and observational studies suggest that acute episodes of heavy alcohol consumption are more strongly related to aggressive behavior than chronic alcohol consumption (Chermack and Blow 2002; Fals-Stewart 2003). This indicates that alcohol-induced aggression is more likely to occur in users who are consuming excessively within a given drinking episode (Heinz et al. 2011), although it is only a minority of people who become aggressive when under the influence of alcohol (Beck and Heinz 2013).

The relation between cannabis use and aggression has also been investigated in studies with animals and with humans. In animals, studies have shown a decrease in aggressive behavior of rodents and primates following cannabis administration (Miczek and Barry 1977; Miczek 1978). In humans, experimental findings on acute effects of cannabis on aggression are mixed (Taylor et al. 1976; Moore and Stuart 2005). Some studies indicate that cannabis intoxication is associated with the elicitation of aggression (Cherek et al. 1993; Howard and Menkes 2007). However, interpretation of these findings is difficult as they are based on relatively small sample sizes (Cherek et al. 1993; Howard and Menkes 2007) or only included male participants with self-reported anti-social tendencies (Cherek et al. 1993). Moreover, dose and route of administration differed considerably between studies. Cannabis effects on aggression are exerted in a dose-dependent manner. Low doses (0.1 mg/kg) of tetrahydrocannabinol (THC) slightly increased the willingness of participants to increase shock intensity given to opponents, were moderate to high doses (0.25–0.4 mg/kg) decreased aggressive response during a laboratory-based aggression study (Myerscough and Taylor 1985). In the latter study, however, participants were randomly assigned to one of the three dose conditions without a placebo condition or control group, making it difficult to assess whether the effect was pharmacological, contextual, or due to individual differences. One study monitored aggression in long-term heavy cannabis users (Kouri et al. 1999) and reported increased aggressive responses relative to controls when tested 3 and 7 days into abstinence.

Aggressive behavior is modulated by neuroendocrine mechanisms, and it is suggested that changes in cortisol and testosterone are predictive of changes in aggression (Brown and Dobs 2002; Brown et al. 2008; Terburg et al. 2009). Fluctuations in cortisol levels can affect the relationship between testosterone and the expression of aggression (Popma et al. 2007). It is unclear whether hormones could play a mediating role in the relationship between drugs and aggression. Previous studies report significant changes in testosterone and cortisol levels following acute alcohol and cannabis administration (Ylikahri et al. 1974; Mendelson et al. 1977; Välimäki et al. 1990; Lovallo et al. 2000; Sarkola et al. 2001; Brown and Dobs 2002; Frias et al. 2002; Thayer et al. 2006). Suppression of male testosterone levels has been reported after short-term heavy drinking (Sarkola and Eriksson 2003), and a reduction in cortisol reactivity was found in heavy drinkers compared to light drinkers after a high (0.8 g/kg) alcohol dose (King et al. 2006). Studies on the effects of cannabis show decreased male testosterone levels after both acute and chronic cannabis use (Kolodny et al. 1974, 1976) and elevated cortisol levels in occasional smokers (Cone et al. 1986); these findings were not corroborated by subsequent studies however (Mendelson et al. 1974; Schaefer et al. 1975; Block et al. 1991).

While aggression is defined objectively as any type of behavior aimed at harming another living being who is motivated to avoid such a behavioral act (Baron 1977), aggression in humans could also be operationalized on a subjective level as the increase in aggressive inclination that is triggered by an aversive/aggressive stimulus or event underlying an emotional-cognitive state. The present study investigated the acute effects of alcohol and cannabis on subjective aggression following aggression exposure in heavy alcohol and regular cannabis users, respectively. Subjective aggression was directly measured by means of a visual analogue scale (VAS) that allowed participants to rate their feeling of aggression on a linear scale ranging from “not aggressive at all” to “very aggressive.” Previous studies (Bond and Lader 1974; Cleare and Bond 1995) that have used rating scales of subjective aggression in human drug studies demonstrated that subjective feelings of aggression and hostility are positively correlated to behavioral acts or measures of aggression. The relevance of subjective aggression therefore lies in the notion that it may predict or coincide with behavioral acts of aggression. Aggression exposure consisted of two tasks developed to evoke and measure aggressive responses: i.e. the single category implicit association test (SC-IAT) and the point-subtraction aggression paradigm (PSAP). Subjective aggression occurring in response to some perceived provocation can be categorized as subjective affective/reactive aggression (Anderson and Bushman 2002). A control group served as between group reference in order to compare aggressive responses of alcohol and cannabis users with non-drug users. Subjective aggression in alcohol and cannabis users was compared with controls while sober and compared to placebo while intoxicated. It was expected that aggression exposure would increase subjective aggression in alcohol users during alcohol intoxication and decrease subjective aggression in cannabis users during cannabis intoxication. It was further expected that subjective aggression would increase after aggression exposure in all groups when sober. Neuroendocrine measures of cortisol and testosterone in response to alcohol/cannabis treatment and after aggression exposure were recorded as additional, secondary outcome measures.

## Experimental procedures

### Participants

The present study included a group of heavy alcohol and regular cannabis users, and a control group. Heavy alcohol use was defined as using on average 21 to 50 alcoholic drinks a week for males or 15 to 35 alcoholic drinks a week for females during the last year (Cassisi et al. 1998). Regular cannabis use was defined as having used cannabis at least 3 times a week but no more than 10 times a week, during the previous year (Ramaekers et al. 2009).

Experimental use of cannabis in the alcohol group was allowed only if it occurred more than a year ago. Alcohol use between 1 and 14 units/week (for both males and females) was allowed in the cannabis group. Controls were defined as not currently using cannabis or other drugs; experimental use of cannabis was allowed if it occurred more than a year ago and incidental alcohol use was permitted (1–7 units/week for women and 1–14 units of alcohol/week for men). Inclusion criteria were as follows: (i) age 18–40 years, (ii) free from psychotropic medication, (iii) good physical health, and (iv) body mass index within 18.5–28 kg/m^2^. Exclusion criteria included: (i) history of drug abuse as assessed by drug urine screens and questionnaires, (ii) presence or history of psychiatric or neurological disorder as assessed by a medical questionnaire, (iii) pregnancy, (iv) cardiovascular abnormalities as assessed by 12-lead ECG, (v) excessive smoking (>15 cigarettes per day) and (vi) hypertension.

Five participants from the alcohol group and 2 participants from the cannabis group dropped out due to personal circumstances. The dropouts were replaced, and the final dataset consisted of 61 participants, i.e. 20 participants in the alcohol and control group and 21 participants in the cannabis group. Participants (35 male, 26 female) were aged between 18 and 28 (mean (SD) 22.5 (2.3) years) (Table 1). Participants’ age in the alcohol and cannabis group were matched with controls. The participants underwent a general medical examination including routine laboratory tests, provided a written informed consent, and filled out a questionnaire on history of drug use. This study was part of a larger experiment and was conducted according to the code of ethics on human experimentation established by the declaration of Helsinki (1964) and amended in Fortaleza (2013) and approved by the Medical Ethics Committee of the Academic Hospital of Maastricht and Maastricht University (Dutch Trial Register: trial number: NTR3428). A permit from the Dutch drug enforcement administration was acquired for obtaining, storing, and administering cannabis. Participants received monetary compensation for their participation in the study.

**Table 1:** Participant demographics and history of alcohol and drug use

	Mean (SD)	Min.	Max.
Age (years) all groups	22.5 (2.3)	18	28
Age alcohol group	22.7 (2.4)	19	28
Age cannabis group	21.9 (2.2)	18	26
Age control group	22.9 (2.3)	19	27
Weight (kg)	67.9 (10.7)	50	92
Alcohol group (*N* = 20; 10 ♂, 10 ♀)			
No. of alcohol units/week	24 (7.7)	15	50
Cannabis group (*N* = 21; 15 ♂, 6 ♀)			
Frequency of cannabis use/week	4.8 (1.9)	3	7
No. of alcohol units/week	4.9 (4.7)	0	14
Control group (*N* = 20; 10 ♂, 10 ♀)			
No. of alcohol units/week	5.3 (3.5)	1	14
Lifetime use of other drugs	Alcohol group	Cannabis group	Control group
Ecstasy	8	10	2
Amphetamine	2	5	1
Cocaine	1	5	0
LSD	0	3	0
Mushrooms	2	11	0
Other (e.g., truffles, ketamine)	3	8	0

*LSD* lysergic acid diethylamide

### Design and treatments

Participants in the alcohol and cannabis group participated in a double-blind, placebo-controlled, within-subject study involving two experimental conditions consisting of placebo and alcohol or cannabis treatment for the alcohol and cannabis group, respectively. An age matched control group of non-drug users was included that received no treatment.

**Fig. 1:**
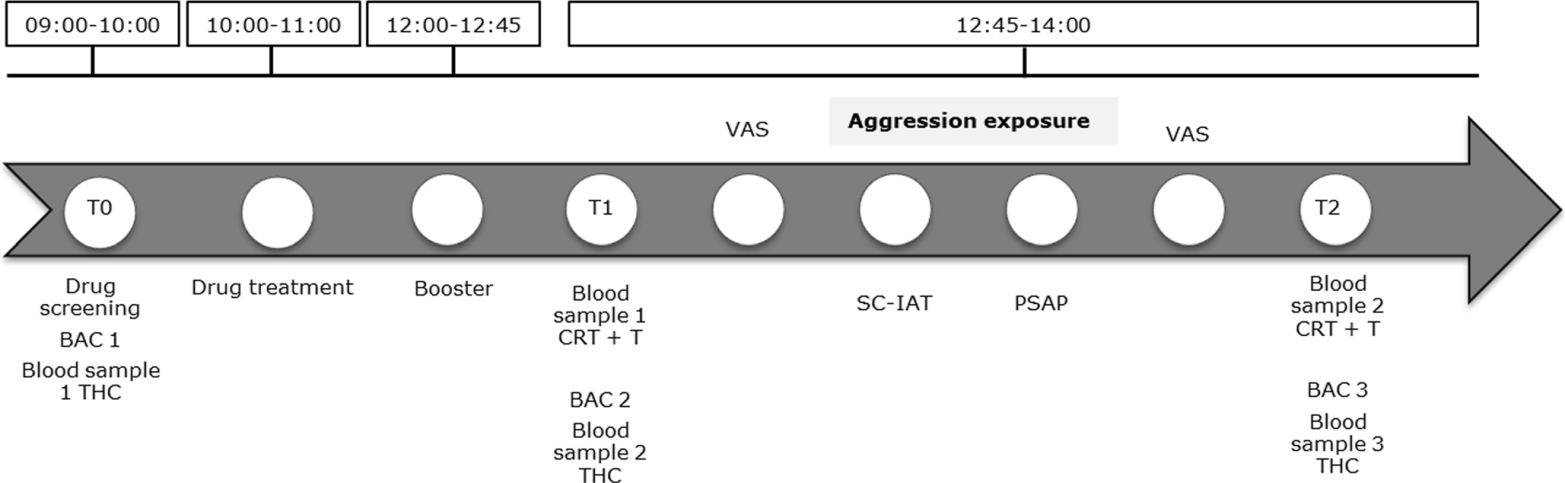
Schematic representation of an experimental session. *BAC* blood alcohol concentration, *THC* tetrahydrocannabinol, *CRT* cortisol, *T* testosterone, *VAS* Visual analogue scale, *SC-IAT* single category implicit association test, *PSAP* point-subtraction aggression paradigm

Alcohol (ethyl alcohol 96 %) was mixed with orange juice to a total volume of 500 mL, divided into two beverages (250 mL each). Alcohol doses were individually calibrated using the formula of Watson et al. (1981) to achieve a total blood alcohol concentration (BAC) of 0.8 g/L, and was kept constant at 0.8 g/L by means of booster doses with an interval of approximately 1 h. The alcohol placebo consisted of 500 mL orange juice, divided into two beverages, which contained a small amount of alcohol (3 mL) to provide an alcohol scent when consuming the beverage. Participants’ BAC was measured at baseline (*T*_0_), before aggression exposure (*T*_1_), and after aggression exposure (*T*_2_) by means of a breathalyzer (Fig. 1).

The cannabis group received in total 300 μg THC/kg bodyweight, divided over two successive doses of 200 and 100 μg THC/kg bodyweight with an interval of approximately 1 h. THC or placebo was administered using a Volcano vaporizer produced by Storz-Bickel, Germany (http://www.storz-bickel.com). Hot air would pass through the filling chamber holding the cannabis (containing 12 % THC), which caused the THC or placebo to vaporize and blend with the air. The THC molecules or the placebo (vapor) was trapped in a valve balloon. The valve of the balloon was put against participants’ lips, and they were instructed to inhale deeply for about 10 s and then exhale. The volume of the balloon was inhaled in 7 to 10 subsequent breaths, and the balloon had to be emptied within 10 min.

### Procedures

Participants were asked to refrain from drug use at least 1 week prior to the start and during the study. Participants were not allowed to use alcohol, tobacco, or caffeinated beverages on the day before an experimental session and were requested to arrive at experimental sessions well-rested. Drug and alcohol screens were carried out upon arrival at our testing facilities. Urine drug screens assessed for the presence of benzodiazepines, opiates, cocaine, marijuana, MDMA, and (meth)amphetamine. Women were also tested for pregnancy. Study treatments were only administered after negative pregnancy tests and negative drug screens, except for marijuana in the cannabis group. For a detailed schematic representation of a test day, see Fig. 1.

The experimental session included an aggression exposure block, which consisted of the administration of the SC-IAT and the PSAP. Subjective aggression was measured before and after aggression exposure. Alcohol (or alcohol placebo) or cannabis (or cannabis placebo) administration was completed at 30 and 15 min prior to aggression exposure, with placebo conditions serving as reference. Conditions were separated by a minimum washout period of 7 days to avoid carry-over effects. The control group received no treatment but the test day was similar on all other aspects. All participants received a training session before onset of the experimental sessions in order to familiarize them with tests, procedures, and in using the vaporizer.

## Assessment of aggression

### Subjective aggression

Subjective aggression was measured using a 100-mm VAS with “not aggressive at all” at one end and “very aggressive” at the other end of the line. Participants had to rate how aggressive they felt at two different time points (i.e., before and after the aggression exposure block). The first time point was aimed to assess the acute effects of alcohol and cannabis treatment on subjective aggression in the alcohol and cannabis group, respectively. The second assessment point followed after a period of aggression manipulation, in which participants were exposed to aggressive stimuli during two laboratory tasks (SC-IAT and PSAP) in order to provoke aggression in the participants. The second time point was aimed to assess both the effects of alcohol and cannabis treatment and the effects of the provocation on subjective aggression.

### Aggression exposure

#### The single category implicit association test

The SC-IAT measures the strength of individuals’ affective evaluative associations (positive vs. negative) with a single attitude object (Greenwald et al. 2003; Karpinski and Steinman 2006). In this task, positive and negative words were coupled with an aggression stimulus. Aggression stimuli were static pictures displaying aggressive acts carried out by other individuals, e.g., violent protests, restrainment with a weapon. Acts of aggression where both physical (i.e., punching, kicking) and verbal (i.e. provocation in traffic-related aggression). The task was divided into a practice block and 2 test blocks (Supplementary Table 1). During the practice block (target discrimination), only the target concepts were presented and participants had to respond using the corresponding keys (i.e. press right button for positive words and the left button for negative words or vice versa). In the first block (compatible block), positive words and aggression stimuli were categorized on the right key and negative words were categorized on the left key. In the second block (incompatible block), negative words and aggression stimuli were categorized on the left key and positive words were categorized on the right key. A correct response was defined when a participant would react to a positive/negative word or an aggression stimulus by pressing the corresponding key. The two blocks were counterbalanced across treatments conditions.

The rationale behind this task is that when participants have a positive association with aggressive behavior rather than a negative association, they are quicker to respond when aggression stimuli are paired up with positive words compared to blocks where aggression stimuli and negative words are paired up. The dependent variable was the D-score, which was calculated by subtracting the mean reaction time (RT) of correct responses in the compatible block from the mean RT of correct responses in the incompatible block, divided by the standard deviation (SD) of all correct responses within the compatible and incompatible block.

#### The point-subtraction aggression paradigm

The PSAP is a free-operant measure of human aggression (Cherek 1981). It is a computer-based task where participants are paired up with a fictitious (unbeknownst to the participant) counterpart and could earn money by pressing buttons. A counter indicating the net value of money earned was shown on the screen. Three response buttons (labeled A, B, or C) were presented to the participants on a row across a response panel: a monetary-reinforced option (A), an aggressive option (B), and an escape option (C). By pressing button A 100 consecutive times, 15 cents was added to the participants’ counter. By pressing button B 10 times, 15 cents was subtracted from the counterpart at no gain to the participant. Button C, the escape option, had to be pressed 10 times and temporarily protected the participants’ money from being subtracted by the fictitious counterpart. Participants were provoked at random intervals throughout the session by having 15 cents subtracted from their counter, which was ostensibly ascribed to the counterpart.

Participants were told that their counterpart was sitting at a different location in the same building. The participants were instructed to earn as much money as possible and had 20 min to complete the task. Participants could freely decide which buttons to press throughout the task and were aware that pressing button C would protect their money for a period of time. Aggression was not mentioned in the instructions. In reality, a computer program controlled all points subtracted by the fictitious counterpart. The dependent variable was the number of times the B button (aggressive responses) had been pressed.

### Neuroendocrine measures

Testosterone and cortisol levels were collected as neuroendocrine measures in response to alcohol and cannabis treatment for the alcohol and cannabis group, respectively, and in response to aggression exposure for all 3 groups. Blood samples (8 mL) to determine cortisol and testosterone concentrations were collected from the participants before (*T*_1_) and after aggression exposure (*T*_2_) (Fig. 1). The blood samples were centrifuged immediately and sent away for analysis after each test day. Concentrations were determined by means of the Cobas assay (Roche Diagnostics Limited, West Sussex, UK). The quantification limit for testosterone and cortisol were 0.087 and 0.500 nmol/L, respectively.

## Pharmacokinetic measures

In the cannabis group, blood samples (8 mL) to determine cannabinoid concentrations (THC and metabolites OH-THC and THC-COOH) were collected at 3 successive times during each test day, i.e. at baseline (*T*_0_) before aggression exposure (*T*_1_) and after aggression exposure (*T*_2_) (Fig. 1). These blood samples were centrifuged immediately; serum was transferred into a tube and was stored at −20 °C. Cannabinoid concentrations were determined by the Institute of Forensic Toxicology, University of Frankfurt, using solid phase extraction and gas chromatography with mass spectrometric detection with a limit of quantification of 1.0 ng/mL.

## Statistics

Two generalized linear models (GLM) were used to analyze differences in subjective aggression and neuroendocrine measures between the 3 groups during abstinence (GLM1) and to test how these measures were affected by acute cannabis and/or alcohol intoxication following aggression exposure compared to placebo (GLM2). VAS data for subjective aggression were log-transformed to obtain a normal distribution.

GLM1 included Group (3 levels; alcohol group when sober, cannabis group when sober, and controls) as the between-subject factor and Aggression exposure (two levels; before and after aggression exposure) as the within-subject factor. These were followed by simple group contrasts with the control group as reference.

GLM2 included Group (2 levels; alcohol and cannabis users) as the between-subject factor and Treatment (2 levels; placebo and alcohol/cannabis) and Aggression exposure (2 levels; before and after aggression exposure) as the within-subjects factors.

The same approach in GLM 1 and 2 was followed for the SC-IAT and PSAP with the exclusion of the factor aggression exposure. In case the sphericity assumption was violated, the Greenhouse-Geisser correction was used. The alpha criterion significance level was set at *p* = 0.05.

Spearman correlations were used to investigate associations between neuroendocrine measures, subjective aggression, and performance in the PSAP and SC-IAT when sober and when intoxicated. All statistical tests were conducted with SPSS version 21.

## Results

### Missing data

A total of 20 complete datasets for the alcohol and control group and 21 datasets for the cannabis group entered the analyses for the SC-IAT. Due to technical failures, complete data sets were missing for the PSAP (4 participants) and aggression VAS (2 participants) PSAP data of one participant was excluded from analysis due to extreme values. Due to difficulties during blood sample collection, testosterone and cortisol samples from 14 participants could not be collected during both experimental sessions (see Supplementary Table 2).

### Measures of aggression

#### GLM 1: comparisons across groups while sober

**Fig. 2:**
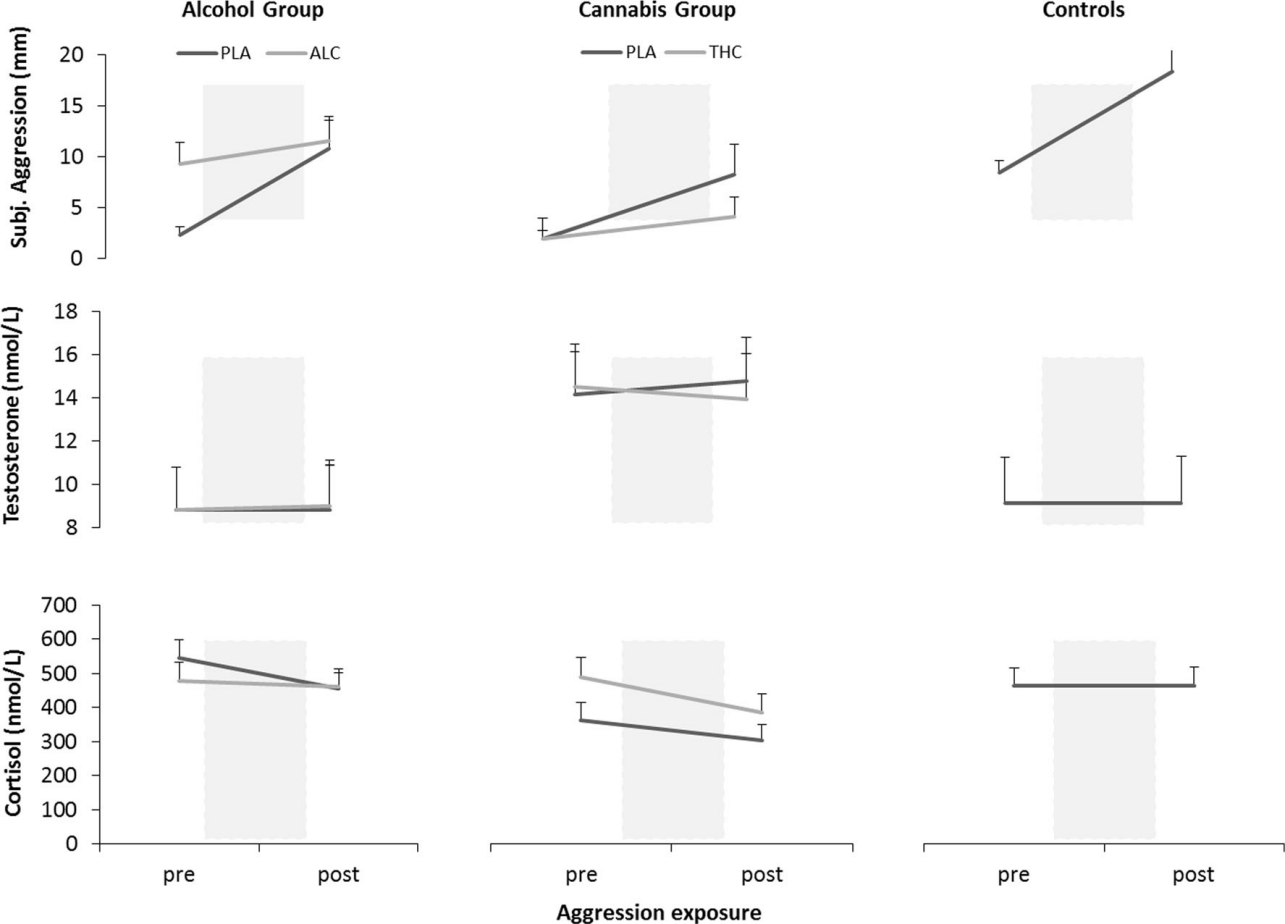
Mean (SE) subjective aggressive ratings (*upper panel*), testosterone concentrations (*middle panel*), cortisol concentrations (*lower panel*) before and after aggression exposure for each group and treatment condition. *PLA* placebo, *ALC* alcohol, *THC* cannabis

GLM analyses revealed a main effect of Aggression exposure on subjective aggression (*F*_2,58_ = 28.31; *p* = .000) when sober. Subjective ratings across groups were higher after aggression exposure compared to before (Fig. 2). There was no effect of Group or interaction with Aggression exposure when sober.

**Fig. 3:**
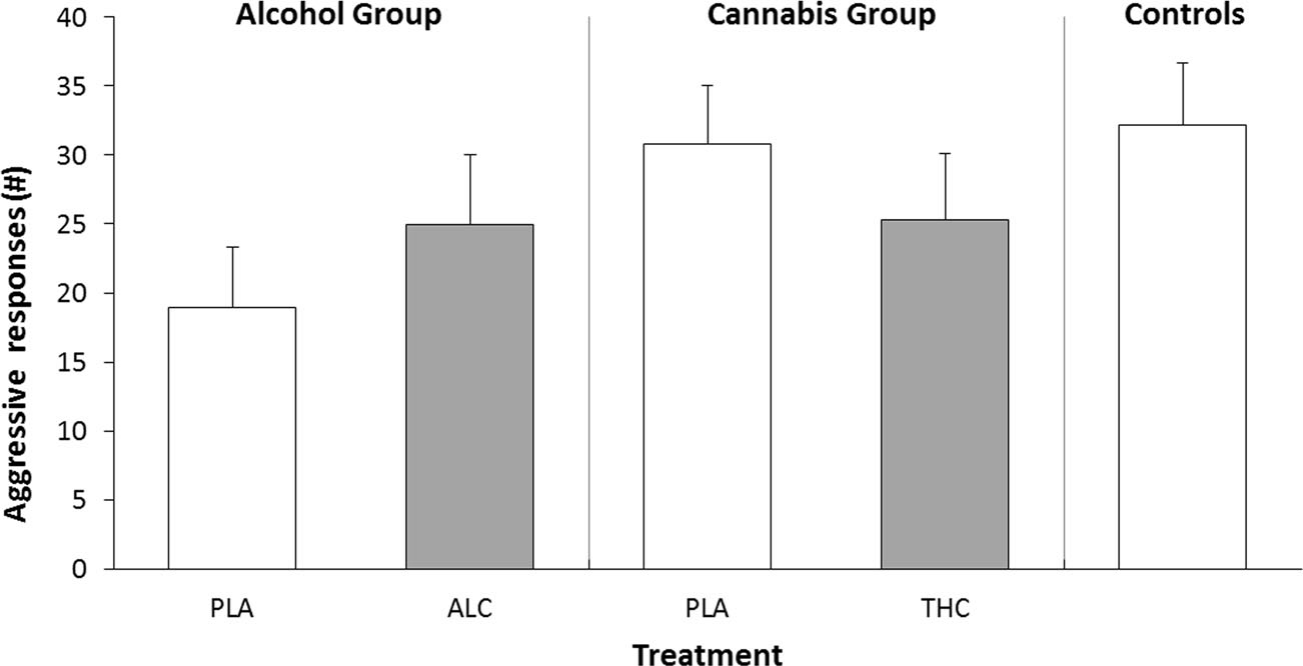
Mean (SE) number of aggressive responses in the point-subtraction aggression paradigm for each group and treatment condition. The number of aggressive responses (B) was equal to 10 button presses. *PLA* placebo, *ALC* alcohol, *THC* cannabis

There was no main difference in aggressive responses between groups during the PSAP when sober (Fig. 3). Escape response rates did differ across the 3 groups during sobriety (*F*_2,53_ = 4.17; *p* = .021). Simple contrast revealed a difference in escape response rates between the control group and alcohol group (*p* = .006), but not between the cannabis group and controls (*p* = .189). Escape response rates in the alcohol group was lower compared to controls. A summary of mean (SE) monetary, aggressive, and escape rates is given in Supplementary Table 3.

Mean D-scores (SE) during the SC-IAT were negative and did not differ between groups when sober (i.e., alcohol (−0.124 (.07)), cannabis (−0.118(.04)), controls (−0.195(.07))).

GLM analyses revealed no main effects of Group or Aggression exposure nor the interaction between Group and Aggression exposure on testosterone levels (Fig. 2). Analyses revealed a main effect of Aggression exposure on cortisol levels (*F*_2,46_ = 6.62; *p* = .013) when sober. Cortisol levels across groups were lower after aggression exposure compared to before. Cortisol levels in the control group before and after exposure actually did not differ significantly from each other (difference score 1.53 nmol/L.) There was no main effect of Group (*F*_2,46_ = 3.09; *p* = .055) or interaction with Aggression exposure on cortisol levels when sober.

There were no significant correlations between neuroendocrine measures, subjective aggression, and performance during the PSAP and SC-IAT in the 3 groups when sober.

#### GLM 2: comparisons between treatments and placebo

GLM analyses revealed a main effect of Aggression exposure (*F*_1,37_ = 17.05; *p* = .000) and Group (*F*_1,37_ = 4.19; *p* = .048) on subjective aggression. Subjective aggression was generally higher after aggression exposure compared to before, but differed between the alcohol and cannabis group. Subjective aggression in the alcohol group was overall higher compared to the cannabis group. Analysis revealed a significant interaction between Treatment and Group (*F*_1,37_ = 7.08; *p* = .011) on subjective aggression. Subjective aggression in the alcohol group was higher in the alcohol compared to placebo condition, whereas subjective aggression in the cannabis group was lower in the cannabis conditions compared to placebo (Fig. 2). There was no effect of Treatment or interaction with Aggression exposure.

There was no effect of Treatment or Group on D-scores during the SC-IAT. Mean D-scores (SE) following alcohol (−0.160 (.07)) and cannabis (−0.229 (.06)) treatment did not differ from placebo conditions or between groups.

GLM analyses revealed no main effects, but a significant interaction between Treatment and group (*F*_1,34_ = 6.16; *p* = .018) on aggressive responses in the PSAP. Aggressive responses in the alcohol group were higher in the alcohol compared to placebo condition, whereas aggressive responses in the cannabis group were lower in the cannabis conditions compared to placebo (Fig. 3). Both monetary response and escape rates (i.e. A responses and C responses) during alcohol or cannabis intoxication did not differ from placebo conditions or between these two groups.

GLM analyses revealed no main effects of Treatment, Aggression exposure, or Group, but a significant 3-way interaction of Treatment*Aggression exposure*Group on testosterone (*F*_1,30_ = 4.92; *p* = .034) and cortisol levels (F_1,31_ = 6.32; *p* = .017) (Fig. 2). The alcohol group did not show a change in testosterone levels after alcohol treatment following aggression exposure compared to placebo. The cannabis group on the other hand showed a decrease in testosterone levels after cannabis treatment, particularly after aggression exposure. Participants in the alcohol group had a small decrease in cortisol levels after alcohol treatment whereas participants in the cannabis group showed an increase in cortisol levels after cannabis treatment, particularly prior to aggression, compared to placebo.

During alcohol and cannabis intoxication, subjective ratings following aggression exposure positively correlated with respectively aggressive responses (*r*_s_(13) = .637; *p* = .011) and escape responses (*r*_s_(18) = .491; *p* = .028) in the PSAP.

There were no significant correlations between neuroendocrine measures and performance during the PSAP and SC-IAT in the alcohol and cannabis group during intoxication.

## Pharmacokinetic measures

Mean alcohol concentrations in blood and cannabinoid concentrations in serum for the alcohol and cannabis treatment conditions from the participants are shown in Table 2.

**Table 2:** Mean (SE) concentrations of THC and metabolites in serum in the cannabis group and blood alcohol concentrations levels in the alcohol group at the different time points

	THC [μg/L]	THC-OH [μg/L]	THC-COOH [μg/L]	BAC [g/L]
Baseline	1.07(.40)	.36(.23)	16.84(1.32)	.00 (.00)
Before aggression measures (12:45)	46.48 (1.59)	3.93 (.26)	27.66 (0.84)	.79 (.02)
After aggression measures (14:00)	24.17 (1.46)	3.16 (.28)	27.34 (1.02)	.60 (.03)

*THC* tetrahydrocannabinol, *THC-OH* 11-hydroxy-THC, *THC-COOH* 11-nor-9-carboxy-THC; *BAC* blood alcohol concentration

## Discussion

The aim of the present study was to assess the acute effects of alcohol and cannabis on subjective aggression in heavy alcohol and regular cannabis users after aggression exposure. Alcohol users received alcohol or placebo and cannabis users were given cannabis or placebo. A group of non-drug users served as controls. Neuroendocrine measure of testosterone and cortisol were collected as additional outcome measures in response to acute alcohol and cannabis intoxication and after aggression exposure.

Subjective aggression after aggression exposure was increased across all 3 groups but did not differ between groups when sober, indicating that the aggression manipulation was successful. Aggressive responses of sober alcohol and cannabis users after aggression exposure did not differ from controls during the PSAP. All groups had equal negative D-scores in the SC-IAT when sober, indicating that all 3 groups had a negative implicit association with aggression. Testosterone levels did not change after aggression exposure or differ between groups. Cortisol levels, on the other hand, decreased to similar degrees in all groups after aggression exposure. Taken together, we did not record any difference in subjective aggression and aggressive responses between sober alcohol and cannabis users and drug-free controls.

Subjective experience of aggression exposure was modified by treatments as compared to placebo, as indicated by significant Group × Treatment interactions. Alcohol intoxication increased subjective aggression in the alcohol group. The cannabis group in contrast experienced a reduction in subjective aggression during cannabis intoxication. Although alcohol intoxication increased subjective feelings of aggression in heavy alcohol users, the general increment was relatively mild. Yet, the direction of the change suggests that alcohol users might feel more aggressive with higher alcohol doses. Likewise, alcohol increased the number of aggressive responses in the PSAP in the alcohol group, whereas cannabis reduced the number of aggressive responses in the cannabis group. These interactions between Treatment and Group point to opposing effects of alcohol and cannabis on aggression. These findings are generally in line with previous studies that showed alcohol-induced aggression at higher doses. A study conducted among healthy male and female social drinkers showed that moderate (0.4 g/kg) to high (0.8 g/kg) alcohol doses do not increase aggression (Gowin et al. 2010), while others showed a dose-related increase in aggression for both genders at the 0.75 and 1.0 g/kg alcohol doses (Duke et al. 2011). The cannabis group received a moderate to high cannabis dose which diminished aggressive responses during intoxication, which is in line with previous findings (Myerscough and Taylor 1985). Subjective measures of aggressive feelings in the alcohol group were positively correlated to performance in the PSAP during intoxication, i.e. aggressive responses increased in the PSAP with increased feelings of aggression, which was not observed in the cannabis group. This indicates that subjective feelings of aggression in heavy alcohol users coincide with behavioral outcome measures of aggression.

Neuroendocrine responses to alcohol and cannabis were very minimal. We observed no main effects of Treatment, Group, and Aggression exposure, but their 3-way interactions reached significance. These indicated that cannabis reduced testosterone levels following aggression exposure whereas alcohol did not. In addition, there were indications that cannabis increased cortisol levels whereas alcohol decreased cortisol, particularly prior to aggression exposure. Attenuated cortisol response in regular alcohol users following a high alcohol dose have been reported before (Cone et al. 1986; King et al. 2006). During sobriety, cortisol levels in the alcohol and cannabis group were decreased after aggression exposure, whereas no changes in testosterone levels were observed. It has been suggested that the elicitation of aggression is related to fluctuations in testosterone and cortisol or their ratio (Popma et al. 2007). More specifically, heightened levels of testosterone are not enough to elicit aggression as sensitivity to punishment and fear are still inhibiting behavior in the presence of high cortisol levels. When high testosterone is combined with low cortisol levels, aggression is not inhibited and could lead to the expression of aggressive behaviors (Terburg et al. 2009). The decrease in cortisol in the alcohol and cannabis group could also be attributed to the passing of time, but this decrease was not seen in controls. Cortisol levels in the control group before and after exposure actually did not significantly differ from each other. Furthermore, a previous study was conducted that analyzed circadian cortisol levels in a group of healthy participants (*N* = 33) to define the parameters of physiological cortisol secretion (Debono et al. 2009). All participants had undergone detailed, 24-h, 20-min, cortisol profiling and serum cortisol levels between 13 and 14 o’ clock were approximately between 244.55–199.50 nmol/L, respectively, indicating a decrease of 45.05 nmol/L in 60 min. In the current study, serum cortisol level decreases in the alcohol (88.81 nmol/L) and cannabis group (56.75 nmol/L) were larger compared to the results of Debono et al. (2009) suggesting that the decrease in cortisol levels after aggression exposure was not exclusively due to the passing of time. In the current study, changes in neuroendocrine measures and alcohol- or cannabis-induced aggression did not significantly correlate, which suggests that both phenomena are unrelated. Future research however in larger samples of both males and females is needed to investigate the relation between intoxicated aggression and associated changes in testosterone and cortisol levels in more detail. A further limitation of the current study is that it did not assess the effect of higher doses of alcohol and cannabis on aggression. As a final limitation, we note that the sample sizes of the current groups may have been too low to detect all potential but small effects of cannabis and alcohol on behavioral measures of aggression.

The results in the present study support the hypothesis that acute alcohol intoxication increases feelings of aggression and that acute cannabis intoxication reduces feelings of aggression following aggression exposure. Future studies examining the drug-aggression relationship should investigate additional variables, such as consumption patterns of alcohol and drug use, different alcohol and/or drug doses, combined with neuroendocrine measures associated with aggression. A multi-causal approach might be more effective in identifying healthy individuals who are particularly at risk of engaging in intoxicated aggression following exposure to aggression.

## Electronic supplementary material

ESM 1 (DOCX 21 kb)
