# Supplementary material for: Subjective aggression during alcohol and cannabis intoxication before and after aggression exposure
Source: Psychopharmacology (Berl). 2016 Jul 15;233:3331–40. doi: 10.1007/s00213-016-4371-1 (PMC4988999; doi:10.1007/s00213-016-4371-1)
Supplement: Supplementary file 1 — (DOCX 21 kb) [file 213_2016_4371_MOESM1_ESM.docx]

**Supplementary material**

**Supplementary table 1**. Schematic representation of the Single Catergory Implicit Association Test.

| Block | Task | Left key | Right key |
| --- | --- | --- | --- |
| 1 | Target discrimination | Negative words | Positive words |
| 2 | Compatible | Negative words | Positive words + aggressive cues |
| 3 | Incompatible | Negative words + aggressive cues | Positive words |

**Supplementary table 2.** Mean (SE) concentrations of testosterone and cortisol during placebo conditions for all 3 groups and drug conditions for the alcohol and cannabis group at the different time points. PLA = placebo, ALC = alcohol, THC = cannabis, CRT = cortisol; T = testosterone

| **Group**  **Hormone** | **Alcohol**  **T** | | **Cannabis**  **T** | | **Alcohol**  **CRT** | | **Cannabis**  **CRT** | | **Control**  **T** | **Control**  **CRT** |
| --- | --- | --- | --- | --- | --- | --- | --- | --- | --- | --- |
| **Conditions** | **PLA [nmol/L]** | **ALC [nmol/L]** | **PLA [nmol/L]** | **THC [nmol/L]** | **PLA [nmol/L]** | **ALC [nmol/L]** | **PLA [nmol/L]** | **THC [nmol/L]** | **(no treatment)** | |
| **Before aggression measures (12:45)** | **8.79(2.00)** | **8.81(1.98)** | **14.12(2.00)** | **14.48(1.98)** | **545.51(51.69)** | **477.00(56.08)** | **361.39(53.29)** | **490.59(57.80)** | **9.06(2.12)** | **463.86(52.25)** |
| *N* | *N = 18* | *N = 19* | *N = 18* | *N = 17* | *N = 18* | *N = 20* | *N = 18* | *N = 17* | *N = 17* | *N = 17* |
| **After aggression measures (14:00)** | **8.83(2.03)** | **9.01(2.08)** | **14.76(2.03)** | **13.93(2.08)** | **456.69(44.82)** | **461.57(53.52)** | **304.64(46.20)** | **385.36(55.17)** | **9.03(2.17)** | **465.39(54.32)** |
| *N* | *N = 17* | *N = 19* | *N = 18* | *N = 18* | *N = 17* | *N = 20* | *N = 18* | *N = 18* | *N = 16* | *N = 16* |

**Supplementary table 3.** Mean (SE) number of monetary and escape responses in the Point Subtraction Aggression Paradigm for each group and treatment condition. One monetary response equals 100 button (A) presses. One aggressive/ escape response equals 10 button B/ C presses. PLA = placebo, TREAT = treatment

|  | **Monetary resonses (A) PLA** | **Monetary resonses (A) TREAT** | **Aggresive responses (B) PLA** | **Aggressive responses (B) TREAT** | **Escape responses (C) PLA** | **Escape resonses (C) TREAT** |
| --- | --- | --- | --- | --- | --- | --- |
| **Alcohol group** | 200.94 (22.74) | 221.44 (24.13) | 18.95 (4.37) | 24.98 (5.07) | 17.34 (4.19) | 17.80 (4.94) |
| **Cannabis group** | 231.36 (21.51) | 240.82 (22.82) | 30.85 (4.14) | 25.32 (4.80) | 26.84 (3.96) | 26.20 (4.67) |
| **Control group** | 205.83 (80.34) | N/A | 32.20 (4.42) | N/A | 34.53 (19.34) | N/A |
